# Biomarker-based depression risk prediction in chronic heart failure patients: an interpretable machine learning approach

**DOI:** 10.3389/fendo.2025.1737713

**Published:** 2025-12-11

**Authors:** Yuxuan Tao, Chenglong Yao, Runjia Liu, Hongfan Qiu, Xinmei Liu, Bingjie Wang, Haixia Li

**Affiliations:** Guang’anmen Hospital, China Academy of Chinese Medical Sciences, Beijing, China

**Keywords:** depression risk prediction, chronic heart failure, machine learning models, apolipoprotein B, triglyceride-glucose index, random forest, SHapley additive explanation

## Abstract

**Background:**

Chronic heart failure (CHF) is frequently complicated by depression, which worsens prognosis but remains underdiagnosed due to symptom overlap and a lack of objective screening tools. Although biomarkers reflecting lipid metabolism, insulin resistance, inflammation, and neuro-immuno-endocrine imbalance have been implicated in both CHF and depression, their predictive value for psychiatric outcomes in CHF patients is unclear.

**Aim:**

This study aimed to develop and validate interpretable machine learning (ML) models for predicting depression risk in CHF patients via the use of clinical and biomarker data.

**Methods:**

We retrospectively enrolled 3, 110 CHF patients admitted between January 2015 and December 2024 at Guang’anmen Hospital. Demographic, clinical, and laboratory indicators, including apolipoprotein B (ApoB), the triglyceride-glucose (TyG) index, and a novel glycated TyG (gTyG) index, were collected. Logistic regression and restricted cubic spline analyses were used to assess dose–response associations between biomarkers and depression. Eight ML algorithms were trained and evaluated, with model interpretability assessed via SHapley Additive exPlanation (SHAP).

**Results:**

Among the 3, 110 patients, 37.3% had comorbid depression. Elevated ApoB and gTyG indices were strongly associated with depression risk in both the unadjusted and fully adjusted models (ApoB Q4 vs. Q1: OR 5.41, 95% CI 3.72–7.87; gTyG Q4 vs. Q1: OR 2.88, 95% CI 1.88–4.41; both P < 0.001), demonstrating clear nonlinear dose–response relationships. The TyG index was associated with depression in the crude analyses but lost significance after adjustment. Among the ML models, the RF model achieved the best performance (AUC 0.933 in training, accuracy 0.814, sensitivity 0.939). SHAP analysis revealed that the ApoB and gTyG indices were the most influential predictors. A user-friendly web application was developed for individualized risk prediction.

**Conclusion:**

This study demonstrated that the ApoB and gTyG index are robust biomarkers for predicting depression risk in CHF patients. The RF model provided the highest predictive accuracy and interpretability, highlighting its potential utility for early risk stratification and targeted intervention. The incorporation of these biomarkers into routine clinical practice may facilitate timely identification and management of depression in CHF patients, ultimately improving patient outcomes.

## 1 Introduction

Chronic heart failure (CHF) represents the terminal stage of multiple cardiovascular diseases and remains a major public health burden worldwide, with persistently high mortality and rehospitalization rates (1–3). In parallel, depression is one of the most common psychiatric comorbidities in CHF patients, with prevalence estimates of 20–40% (4, 5). This comorbidity significantly aggravates prognosis, leading to poorer treatment adherence, diminished quality of life, and increased mortality. Despite its clinical significance, depression often remains underrecognized and undertreated in CHF because of symptom overlap, atypical presentations, and the lack of objective, time-efficient screening tools (6).

Traditional diagnostic strategies, mainly based on psychometric scales or interviews, are limited by subjectivity and low biological specificity. While predictive models in CHF have largely focused on mortality, readmission, and disease progression, psychiatric outcomes have received insufficient attention. A growing body of evidence has implicated biological pathways such as inflammation, metabolic dysregulation, oxidative stress, and neuro–immune–endocrine imbalance in the pathogenesis of depression in cardiovascular populations (7–9). Biomarkers reflecting these processes may provide a more objective and scalable approach for risk stratification.

However, existing biomarker studies remain preliminary and rarely integrated into predictive frameworks for psychiatric comorbidity. In translational mental health research, there is a critical need to move beyond descriptive associations toward clinically applicable, interpretable tools that can support early intervention. This aligns closely with the translational psychiatry paradigm, which emphasizes mechanistic insight and real-world applicability.

Our study aimed to develop and validate an interpretable machine learning (ML) model to predict the risk of depression among patients with CHF using routinely collected clinical features and biomarkers associated with depression. In this retrospective study, patients with CHF were enrolled. On the basis of both routinely measured clinical indicators and depression-related biological mechanisms, we included biomarkers associated with lipid metabolism, inflammation, oxidative stress and neuro-immunoendocrine imbalance. The associations between these biomarkers and the risk of depression in CHF patients were evaluated via multiple analytical models. The optimal ML model for predicting depression onset in CHF patients was subsequently identified, and a corresponding predictive software tool was developed.

## 2 Materials and methods

### 2.1 Study population

We retrospectively selected patients with CHF who were admitted to Guang’anmen Hospital, China Academy of Chinese Medical Sciences, between January 2015 and December 2024.A total of 8, 645 patients were initially identified from the multimodal clinical data system.

All patients provided written informed consent. The study protocol was approved by the Ethics Committee of Guang’anmen Hospital, China Academy of Chinese Medical Sciences, and was registered with the International Traditional Medicine Clinical Trial Registry (ITMCTR) (Registration number: ITMCTR2025001576).

### 2.2 Diagnostic, inclusion and exclusion criteria

CHF diagnosis followed the ESC Guidelines for the Diagnosis and Treatment of Acute and Chronic Heart Failure 2012 and the Chinese Guidelines for the Diagnosis and Treatment of Heart Failure 2014; key symptoms/signs, imaging, natriuretic peptides, and echocardiography were used per guidance (10, 11). Eligible patients were aged 18–85 years with confirmed chronic heart failure.

Patients with severe hepatic or renal dysfunction (eGFR <30 mL/min/1.73 m² or requiring dialysis), active autoimmune or infectious diseases affecting metabolic biomarkers, or incomplete clinical or laboratory data required for analysis were excluded from the study. For patients with multiple admissions, only the first admission was retained. Following the application of these criteria, the analysis was conducted on a total of 3, 110 patients.

### 2.3 Data collection and variables

From electronic records we extracted demographics/vitals, comorbidities, and laboratory measures (hematologic, biochemical, inflammatory/coagulation panels). Derived indices included TyG = ln[TG (mg/dL) × FPG (mg/dL)/2]. We further developed a novel modified index, termed the gTyG index, which is calculated as ln [TG (mg/dL) × HbA1c (mg/dL)/2]. Missingness <20% was imputed via k-nearest neighbors; variables with >20% missingness were excluded.

### 2.4 Definitions of comorbidities and conditions

Defined according to recommendations for screening for depression in adults (12). Core feature: depressed mood, plus ≥4 of the following: loss of interest/pleasure, fatigue, psychomotor retardation or agitation, low self-esteem/guilt, impaired concentration, recurrent suicidal thoughts or behaviors, sleep disturbance, appetite/weight loss, and decreased libido.

Other conditions followed authoritative guidelines: peptic ulcer (13), bradycardia and pacing criteria (14), atrial fibrillation (15), ventricular arrhythmias (16), hypertension (17),, coronary heart disease (18), diabetes (19), stroke (20), pneumonia (21), chronic kidney disease (22), liver disease (23), rheumatoid arthritis (24), asthma (25), thyroid dysfunction (26), osteoporosis (27), and anemia (28).

### 2.5 Statistical analysis

All analyses were performed using SPSS 26.0, R 4.3.3, and Zstats. Normality was assessed with the Kolmogorov–Smirnov (K-S) test. Patients were classified by the presence of comorbidities (0/1) and stratified by sex (male = 1, female = 2). Continuous variables were analyzed with t-tests or Mann–Whitney U tests, and categorical variables with χ² or Fisher’s exact tests. Missing data were imputed by k-nearest neighbor for variables with <20% missingness; variables with >20% missingness were excluded. Multivariable logistic regression was used to estimate odds ratios across quartiles of ApoB, TyG, and gTyG in three progressively adjusted models. Restricted cubic splines evaluated nonlinear dose–response relationships. Subgroup analyses tested the robustness of these associations. All tests were two-sided, with statistical significance set at P < 0.05.

### 2.6 Model development, evaluation, and interpretability

Data were split 70/30 into training/testing sets. Eight ML classifiers, including artificial neural network (ANN), decision tree (DT), gradient boosting machine (GBM), K-nearest neighbors (KNN), LightGBM (LGBM), random forest (RF), support vector machine (SVM) and XGBoost (XGB), were tuned by grid search with cross-validation. Discrimination and classification performance were summarized by ROC-AUC, accuracy, sensitivity, specificity, positive predictive value (PPV), negative predictive value (NPV), F1 score, and Cohen’s kappa coefficient. SHapley Additive exPlanation (SHAP) quantified global and local feature contributions. A lightweight Flask web interface was prototyped for bedside use.

## 3 Results

### 3.1 Patient selection

From the multimodal data system, a total of 8, 645 patients with CHF admitted between January 2015 and December 2024 were initially identified. Patients were excluded if they were missing >20% laboratory data (n=2, 598), aged <18 or >85 years (n=826), had severe liver or renal disease (n=525), or had active inflammatory/rheumatologic diseases affecting the ApoB, IR, or TyG indices (n=383). For patients with multiple hospitalizations, only the first admission was included (n=1, 203). Ultimately, 3, 110 patients were enrolled for cohort analysis (**Supplementary Figure 1****).**

### 3.2 Baseline characteristics

Among 3, 110 CHF patients, 1, 552 (49.90%) were male and 1, 558 (50.10%) were female, with a median age of 68 years (IQR: 60–75). A total of 1, 159 patients (37.27%) were classified into the depression group, and 1, 951 (62.73%) were included in the control group. The depression group showed significantly higher levels of ApoB, TyG, gTyG, Age, T, CHO, CH50, SG, Cl, TG, A/G, DBIL, RDWSD, FDP, PLT, and HGB and higher prevalence of hypertension, CHD, diabetes, stroke, OP, and anemia (all P < 0.05). Conversely, HR, DBP, Scr, UA, TP, LYMPH, GLB, ALB, ALP, P, RBC, HCT, FIB, CHE, and PCT were lower (all P < 0.05) (**Table 1****).** These findings suggest that ApoB, TyG, and gTyG are strongly associated with depressive status in CHF patients, supporting further dose–response analyses.

**Table 1:** Baseline characteristics.

Variables	Total (n = 3110)	Control (n = 1951)	Depression (n = 1159)	Statistic	P
ApoB, M (Q_1_, Q_3_)	0.78 (0.64, 0.97)	0.73 (0.59, 0.86)	0.92 (0.78, 1.10)	Z=-20.12	**<.001**
TyG, M (Q_1_, Q_3_)	1.33 (1.06, 1.70)	1.28 (1.01, 1.64)	1.41 (1.15, 1.78)	Z=-7.79	**<.001**
gTyG, M (Q_1_, Q_3_)	1.39 (1.18, 1.69)	1.37 (1.11, 1.61)	1.48 (1.29, 1.81)	Z=-9.79	**<.001**
Age, M (Q_1_, Q_3_)	68.00 (60.00, 75.00)	67.00 (59.00, 75.00)	69.00 (61.00, 77.00)	Z=-3.92	**<.001**
T, M (Q_1_, Q_3_)	36.40 (36.20, 36.50)	36.40 (36.20, 36.50)	36.40 (36.20, 36.50)	Z=-2.21	**0.027**
HR, M (Q_1_, Q_3_)	74.00 (67.00, 80.00)	74.00 (67.00, 81.00)	72.00 (66.00, 80.00)	Z=-3.41	**<.001**
R, M (Q_1_, Q_3_)	18.00 (18.00, 19.00)	18.00 (18.00, 19.00)	18.00 (18.00, 19.00)	Z=-0.33	0.738
SBP, M (Q_1_, Q_3_)	135.00 (122.00, 148.00)	135.00 (122.00, 148.00)	135.00 (122.00, 148.00)	Z=-0.39	0.699
DBP, M (Q_1_, Q_3_)	78.00 (69.00, 85.00)	78.00 (70.00, 85.00)	76.00 (68.00, 83.00)	Z=-3.82	**<.001**
LDH, M (Q_1_, Q_3_)	163.00 (144.00, 186.00)	163.00 (144.00, 186.00)	163.00 (145.00, 186.00)	Z=-0.30	0.763
TT, M (Q_1_, Q_3_)	15.70 (14.70, 16.80)	15.70 (14.70, 16.80)	15.70 (14.70, 16.70)	Z=-1.25	0.213
PA, M (Q_1_, Q_3_)	22.25 (19.40, 25.30)	22.25 (19.50, 25.41)	22.25 (19.24, 25.00)	Z=-1.60	0.109
PLCR, M (Q_1_, Q_3_)	27.20 (22.80, 32.00)	27.20 (22.60, 32.00)	27.20 (23.30, 31.90)	Z=-0.75	0.452
SRC, M (Q_1_, Q_3_)	1.20 (0.60, 2.70)	1.20 (0.60, 2.80)	1.20 (0.60, 2.50)	Z=-2.16	**0.031**
UA, M (Q_1_, Q_3_)	314.00 (253.25, 374.75)	319.00 (257.00, 383.50)	307.00 (247.00, 362.50)	Z=-4.63	**<.001**
MPV, M (Q_1_, Q_3_)	10.30 (9.80, 10.90)	10.30 (9.80, 10.90)	10.30 (9.80, 10.90)	Z=-0.77	0.440
MCH, M (Q_1_, Q_3_)	30.60 (29.80, 31.40)	30.60 (29.70, 31.40)	30.60 (29.80, 31.50)	Z=-0.93	0.354
MCHC, M (Q_1_, Q_3_)	336.00 (330.00, 342.00)	336.00 (330.00, 341.00)	336.00 (330.00, 342.00)	Z=-0.07	0.944
CHO, M (Q_1_, Q_3_)	4.15 (3.43, 4.88)	4.34 (3.75, 5.14)	3.69 (3.12, 4.34)	Z=-16.66	**<.001**
TBIL, M (Q_1_, Q_3_)	12.90 (10.50, 16.00)	12.90 (10.50, 15.90)	12.90 (10.40, 16.05)	Z=-0.08	0.939
TP, M (Q_1_, Q_3_)	66.20 (62.50, 70.00)	66.60 (62.90, 70.40)	65.30 (61.60, 69.40)	Z=-5.23	**<.001**
CH50, M (Q_1_, Q_3_)	60.30 (52.10, 65.90)	60.30 (52.80, 66.40)	60.30 (51.30, 65.20)	Z=-3.45	**<.001**
Nlrbc, M (Q_1_, Q_3_)	2.90 (1.70, 5.00)	2.90 (1.70, 5.20)	2.90 (1.80, 4.80)	Z=-0.48	0.633
SG, M (Q_1_, Q_3_)	1.01 (1.01, 1.02)	1.01 (1.01, 1.02)	1.01 (1.01, 1.02)	Z=-2.43	**0.015**
Cl, M (Q_1_, Q_3_)	105.20 (103.20, 106.90)	105.10 (103.10, 106.80)	105.40 (103.30, 107.20)	Z=-3.31	**<.001**
APTT, M (Q_1_, Q_3_)	25.80 (24.30, 27.30)	25.80 (24.30, 27.30)	25.80 (24.20, 27.50)	Z=-0.03	0.979
Aamy, M (Q_1_, Q_3_)	59.00 (50.00, 69.00)	59.00 (50.00, 69.00)	59.00 (49.00, 69.00)	Z=-0.18	0.855
LYMPH, M (Q_1_, Q_3_)	1.57 (1.27, 1.92)	1.57 (1.31, 1.95)	1.57 (1.24, 1.86)	Z=-3.56	**<.001**
GLB, M (Q_1_, Q_3_)	26.90 (24.46, 29.92)	27.20 (24.61, 30.30)	26.57 (24.10, 29.30)	Z=-4.55	**<.001**
TG, M (Q_1_, Q_3_)	1.31 (1.04, 1.65)	1.31 (1.14, 1.79)	1.31 (0.96, 1.43)	Z=-10.14	**<.001**
A/G, M (Q_1_, Q_3_)	1.45 (1.29, 1.61)	1.44 (1.28, 1.60)	1.46 (1.31, 1.61)	Z=-2.59	**0.010**
WBC, M (Q_1_, Q_3_)	6.04 (5.17, 7.01)	6.04 (5.21, 7.06)	6.04 (5.11, 6.94)	Z=-1.26	0.207
ALB, M (Q_1_, Q_3_)	39.00 (36.74, 41.43)	39.10 (36.90, 41.60)	38.73 (36.47, 41.20)	Z=-3.04	**0.002**
DBIL, M (Q_1_, Q_3_)	2.70 (2.00, 3.60)	2.60 (2.00, 3.50)	2.80 (2.10, 3.80)	Z=-4.23	**<.001**
ALP, M (Q_1_, Q_3_)	70.00 (60.00, 82.00)	70.00 (60.50, 83.00)	70.00 (59.00, 82.00)	Z=-2.32	**0.020**
P, M (Q_1_, Q_3_)	1.19 (1.07, 1.31)	1.20 (1.08, 1.32)	1.18 (1.07, 1.31)	Z=-2.04	**0.041**
NSE, M (Q_1_, Q_3_)	10.83 (9.64, 12.40)	10.83 (9.71, 12.56)	10.83 (9.57, 12.29)	Z=-1.70	0.089
CASTM, M (Q_1_, Q_3_)	0.13 (0.00, 0.15)	0.13 (0.00, 0.15)	0.13 (0.00, 0.14)	Z=-1.76	0.078
HbA1c, M (Q_1_, Q_3_)	6.10 (5.70, 6.70)	6.10 (5.70, 6.70)	6.10 (5.70, 6.60)	Z=-0.61	0.540
RBC, M (Q_1_, Q_3_)	4.27 (3.94, 4.59)	4.27 (3.97, 4.62)	4.27 (3.91, 4.54)	Z=-3.00	**0.003**
RDWCV, M (Q_1_, Q_3_)	12.70 (12.30, 13.20)	12.70 (12.30, 13.20)	12.70 (12.30, 13.20)	Z=-1.67	0.094
RDWSD, M (Q_1_, Q_3_)	42.30 (40.60, 44.40)	42.30 (40.50, 44.30)	42.30 (40.80, 44.50)	Z=-1.97	**0.049**
HCT, M (Q_1_, Q_3_)	38.70 (35.80, 41.40)	38.70 (36.00, 41.70)	38.70 (35.45, 40.90)	Z=-2.90	**0.004**
MCV, M (Q_1_, Q_3_)	90.70 (88.60, 92.97)	90.70 (88.40, 92.90)	90.70 (89.00, 93.00)	Z=-1.39	0.166
FIB, M (Q_1_, Q_3_)	3.00 (2.57, 3.54)	3.00 (2.61, 3.60)	2.99 (2.53, 3.44)	Z=-3.18	**0.001**
FDP, M (Q_1_, Q_3_)	2.60 (2.50, 2.60)	2.60 (2.50, 2.60)	2.60 (2.50, 2.60)	Z=-2.05	**0.041**
CK, M (Q_1_, Q_3_)	71.50 (51.25, 99.75)	71.50 (51.00, 100.00)	71.50 (52.00, 98.50)	Z=-0.17	0.867
CHE, M (Q_1_, Q_3_)	7.19 (6.17, 8.23)	7.24 (6.32, 8.39)	7.06 (6.01, 7.88)	Z=-6.29	**<.001**
PLT, M (Q_1_, Q_3_)	205.00 (177.00, 239.00)	205.00 (180.00, 243.00)	205.00 (172.00, 236.00)	Z=-2.88	**0.004**
PDW, M (Q_1_, Q_3_)	11.60 (10.60, 12.80)	11.60 (10.50, 12.80)	11.60 (10.70, 12.80)	Z=-0.49	0.624
PCT, M (Q_1_, Q_3_)	0.21 (0.19, 0.24)	0.21 (0.19, 0.25)	0.21 (0.18, 0.24)	Z=-2.54	**0.011**
HCY, M (Q_1_, Q_3_)	12.60 (10.40, 15.67)	12.60 (10.40, 15.80)	12.60 (10.35, 15.45)	Z=-1.38	0.167
HGB, M (Q_1_, Q_3_)	131.00 (120.00, 140.00)	131.00 (120.00, 141.00)	131.00 (118.00, 138.00)	Z=-3.02	**0.003**
PH, M (Q_1_, Q_3_)	6.00 (5.50, 6.50)	6.00 (5.50, 6.50)	6.00 (5.50, 6.50)	Z=-0.67	0.504
Na, M (Q_1_, Q_3_)	140.00 (138.30, 141.40)	140.00 (138.30, 141.40)	140.10 (138.45, 141.50)	Z=-1.70	0.090
Mg, M (Q_1_, Q_3_)	0.89 (0.84, 0.94)	0.89 (0.85, 0.94)	0.89 (0.84, 0.94)	Z=-1.17	0.241
GENDER, n(%)				χ²=0.39	0.534
1	1552 (49.90)	982 (50.33)	570 (49.18)		
2	1558 (50.10)	969 (49.67)	589 (50.82)		
PU, n(%)				χ²=0.01	0.938
0	3061 (98.42)	1920 (98.41)	1141 (98.45)		
1	49 (1.58)	31 (1.59)	18 (1.55)		
BC, n(%)				χ²=1.72	0.190
0	2941 (94.57)	1853 (94.98)	1088 (93.87)		
1	169 (5.43)	98 (5.02)	71 (6.13)		
AF, n(%)				χ²=0.03	0.857
0	2613 (84.02)	1641 (84.11)	972 (83.87)		
1	497 (15.98)	310 (15.89)	187 (16.13)		
Arrhythmia, n(%)				χ²=1.58	0.208
0	2265 (72.83)	1436 (73.60)	829 (71.53)		
1	845 (27.17)	515 (26.40)	330 (28.47)		
Cancer, n(%)				χ²=1.20	0.273
0	2788 (89.65)	1758 (90.11)	1030 (88.87)		
1	322 (10.35)	193 (9.89)	129 (11.13)		
Hypertension, n(%)				χ²=107.34	**<.001**
0	840 (27.01)	651 (33.37)	189 (16.31)		
1	2270 (72.99)	1300 (66.63)	970 (83.69)		
CHD, n(%)				χ²=13.48	**<.001**
0	775 (24.92)	529 (27.11)	246 (21.23)		
1	2335 (75.08)	1422 (72.89)	913 (78.77)		
Diabetes, n(%)				χ²=9.75	**0.002**
0	1892 (60.84)	1228 (62.94)	664 (57.29)		
1	1218 (39.16)	723 (37.06)	495 (42.71)		
Stroke, n(%)				χ²=24.77	**<.001**
0	1914 (61.54)	1266 (64.89)	648 (55.91)		
1	1196 (38.46)	685 (35.11)	511 (44.09)		
Pneumonia, n(%)				χ²=0.24	0.621
0	2796 (89.90)	1750 (89.70)	1046 (90.25)		
1	314 (10.10)	201 (10.30)	113 (9.75)		
CKD, n(%)				χ²=1.86	0.173
0	2987 (96.05)	1881 (96.41)	1106 (95.43)		
1	123 (3.95)	70 (3.59)	53 (4.57)		
Liver disease, n(%)				χ²=0.98	0.323
0	3096 (99.55)	1944 (99.64)	1152 (99.40)		
1	14 (0.45)	7 (0.36)	7 (0.60)		
RA, n(%)				χ²=0.02	0.891
0	3047 (97.97)	1912 (98.00)	1135 (97.93)		
1	63 (2.03)	39 (2.00)	24 (2.07)		
Asthma, n(%)				χ²=0.89	0.345
0	3038 (97.68)	1902 (97.49)	1136 (98.02)		
1	72 (2.32)	49 (2.51)	23 (1.98)		
TD, n(%)				χ²=0.67	0.414
0	1843 (59.26)	1167 (59.82)	676 (58.33)		
1	1267 (40.74)	784 (40.18)	483 (41.67)		
OP, n(%)				χ²=7.22	**0.007**
0	2867 (92.19)	1818 (93.18)	1049 (90.51)		
1	243 (7.81)	133 (6.82)	110 (9.49)		
Anemia, n(%)				χ²=5.22	**0.022**
0	2732 (87.85)	1734 (88.88)	998 (86.11)		
1	378 (12.15)	217 (11.12)	161 (13.89)		

Z: Mann—Whitney test, χ^2^: Chi-square test.

M: Median, Q_1_: 1st Quartile, Q_3_: 3 st Quartile. Bold P values indicate statistical significance at P < 0.05.

### 3.3 Associations of ApoB, gTyG, and TyG with depression

**Figure 1:**
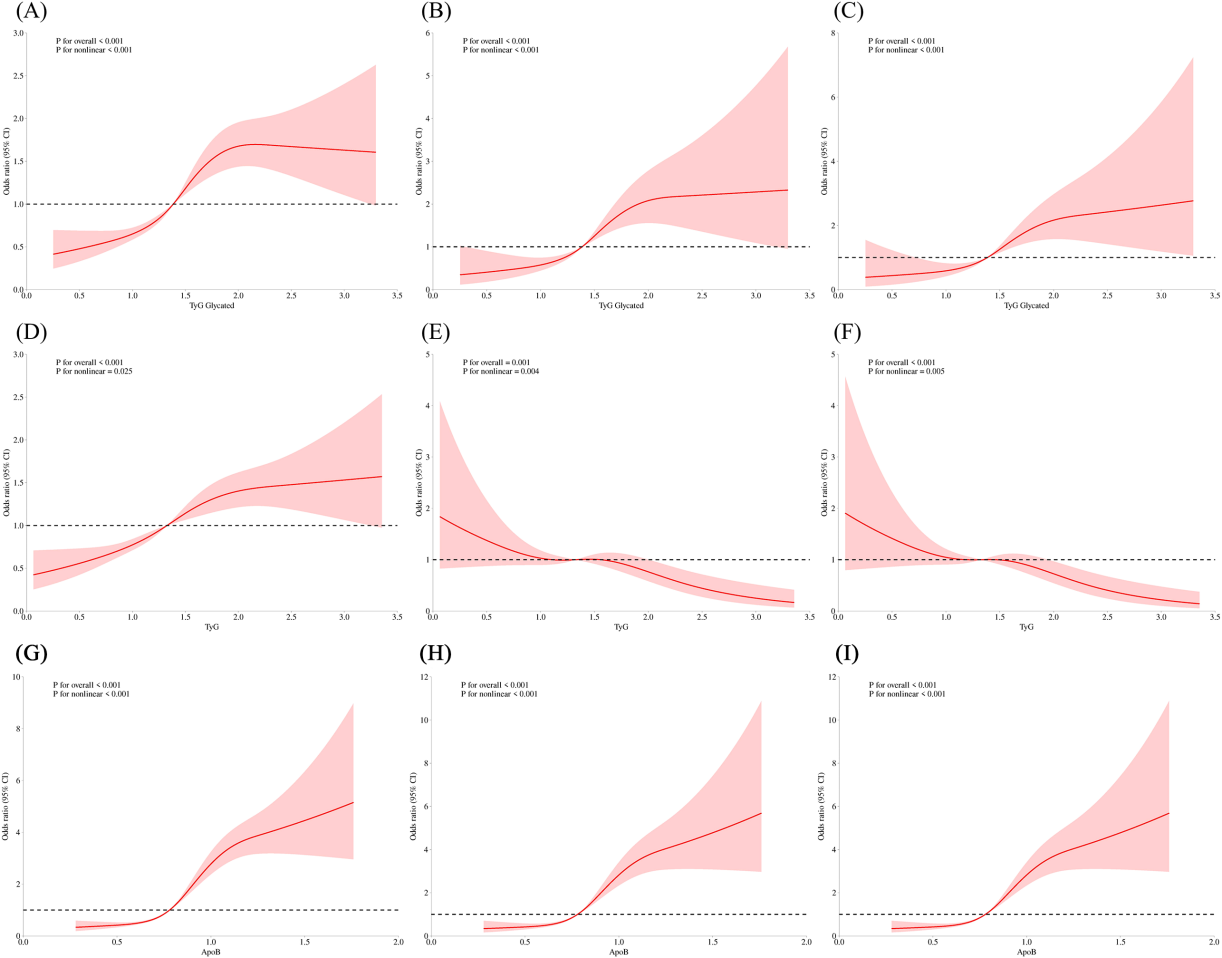
Restricted cubic spline (RCS) curves of ApoB, gTyG index and TyG index levels across different regression models. **(A)** RCS curve of gTyG index levels in the unadjusted model. **(B)** RCS curve of gTyG index levels in the partially adjusted model. **(C)** RCS curve of gTyG index levels in the fully adjusted model. **(D)** RCS curve of TyG index levels in the unadjusted model. **(E)** RCS curve of TyG index levels in the partially adjusted model. **(F)** RCS curve of TyG index levels in the fully adjusted model. **(G)** RCS curve of ApoB levels in the unadjusted model. **(H)** RCS curve of ApoB levels in the partially adjusted model. **(I)** RCS curve of ApoB levels in the fully adjusted model.

Using ApoB quartiles as the independent variable, logistic regression showed that compared with Q1, the risk ratios for depression were 1.53, 3.00, and 7.90 in Q2–Q4 in the unadjusted model, and 1.26, 2.26, and 5.41 in the fully adjusted model (all P < 0.001 for Q3–Q4). Restricted cubic spline analyses further revealed significant nonlinear associations between ApoB and depression risk in both unadjusted and adjusted models (P for nonlinearity < 0.001) (**Figures 1G–I**, **Table 2**).

**Table 2:** Risk ratios of depression across ApoB levels in different models.

Variables	Model1		Model2		Model3	
	OR (95%CI)	P	OR (95%CI)	P	OR (95%CI)	P
ApoB group						
1	1.00 (Reference)		1.00 (Reference)		1.00 (Reference)	
2	1.53 (1.21 ~ 1.94)	**<.001**	1.31 (1.01 ~ 1.71)	**0.048**	1.26 (0.97 ~ 1.65)	0.089
3	3.00 (2.38 ~ 3.78)	**<.001**	2.38 (1.75 ~ 3.22)	**<.001**	2.26 (1.66 ~ 3.08)	**<.001**
4	7.90 (6.26 ~ 9.95)	**<.001**	5.81 (4.01 ~ 8.41)	**<.001**	5.41 (3.72 ~ 7.87)	**<.001**

OR: Odds Ratio, CI: Confidence Interval.

Model1: Crude.

Model2: Adjust: TyG index, gTyG index, Age, HR, DBP, UA, CHO, TP, CH50, Cl, LYMPH, GLB, DBIL, CHE.

Model3: Adjust: TyG index, gTyG index, Age, T, HR, DBP, SRC, UA, CHO, TP, CH50, SG, Cl, LYMPH, GLB, A/G, ALB, DBIL, ALP, P, RBC, RDWSD, HCT, FIB, FDP, CHE, PLT, PCT, HGB.

Bold P values indicate statistical significance at P < 0.05.

Similarly, for gTyG, the risk ratios were 1.65, 1.99, and 2.81 in the unadjusted model, and 1.48, 1.93, and 2.88 in the fully adjusted model (P < 0.001 for Q3–Q4). Nonlinear associations were also observed (P for nonlinearity < 0.001) (**Figures 1A–C**, **Supplementary Table 1**).

For TyG, the unadjusted model showed significant risk elevation across quartiles (RR 1.55, 1.91, and 2.14; all P < 0.001), but after adjusting for covariates, the association was no longer significant (Q4 vs Q1: P = 0.658). Nevertheless, nonlinear patterns persisted (P for nonlinearity = 0.025 and 0.005 in Model 1 and 3, respectively), suggesting confounding effects (**Figures 1D–F**, **Supplementary Table 2**).

### 3.4 Subgroup analyses

**Figure 2:**
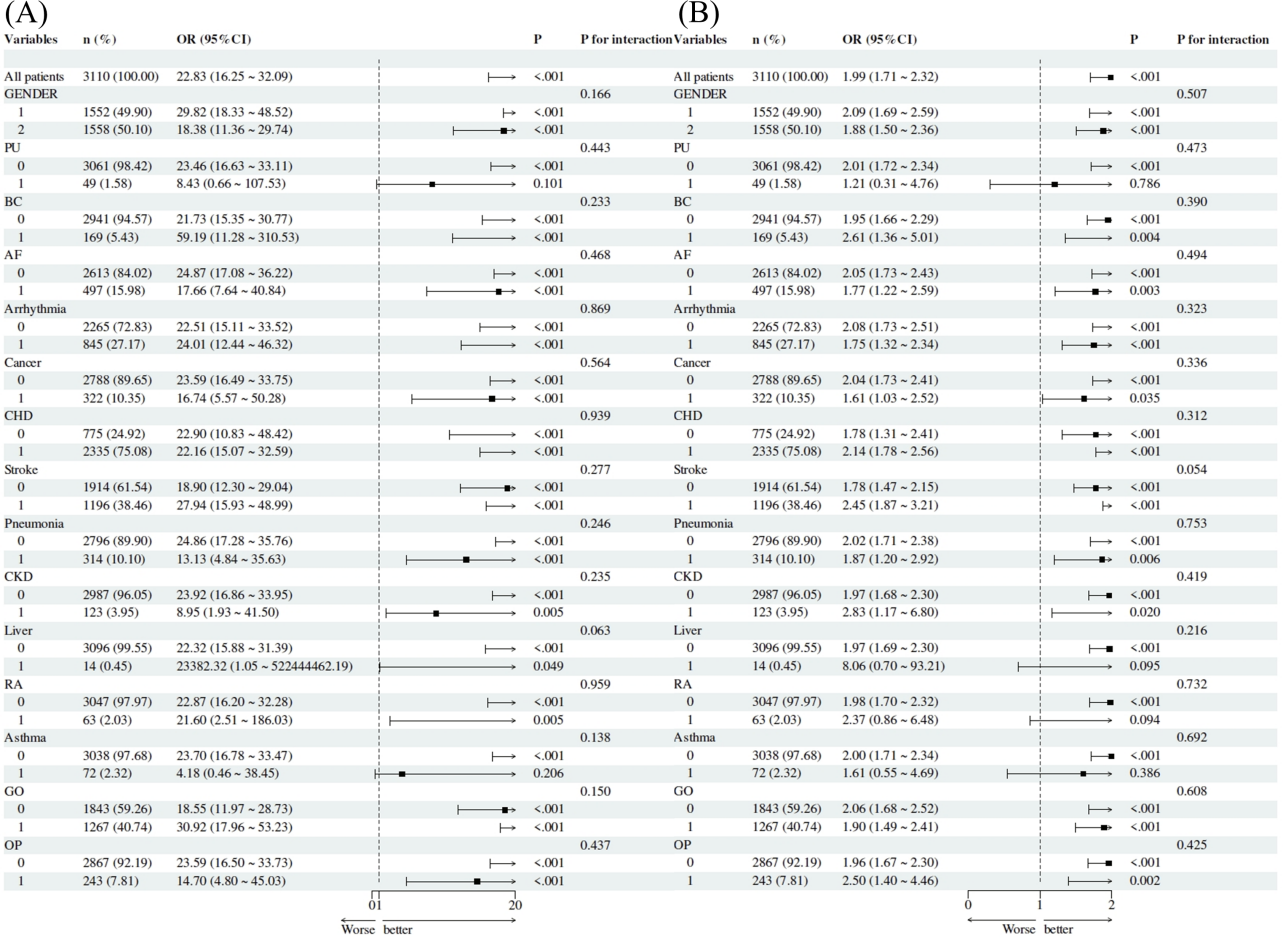
Subgroup analyses of the associations among ApoB, the gTyG index, and depression in CHF patients. **(A)** ApoB levels. **(B)** gTyG index levels. Adjusted ORs (95% CIs) are shown across predefined subgroups, with consistent associations and no significant interactions.

To further evaluate the robustness of the associations between ApoB, the gTyG index, and depression risk, subgroup analyses were conducted using depression status in CHF patients (coded: no = 0, yes = 1) as the dependent variable and ApoB and gTyG index levels as independent variables. The results revealed that ApoB was significantly associated with depression risk across multiple subgroups, including sex, BC, AF, arrhythmia, cancer, CHD, diabetes, stroke, pneumonia, CKD, TD, and OP (all P < 0.05). No significant interaction was detected in any subgroup (all P values for interaction > 0.05) (**Figure 2**).

### 3.5 Model development and performance

**Figure 3:**
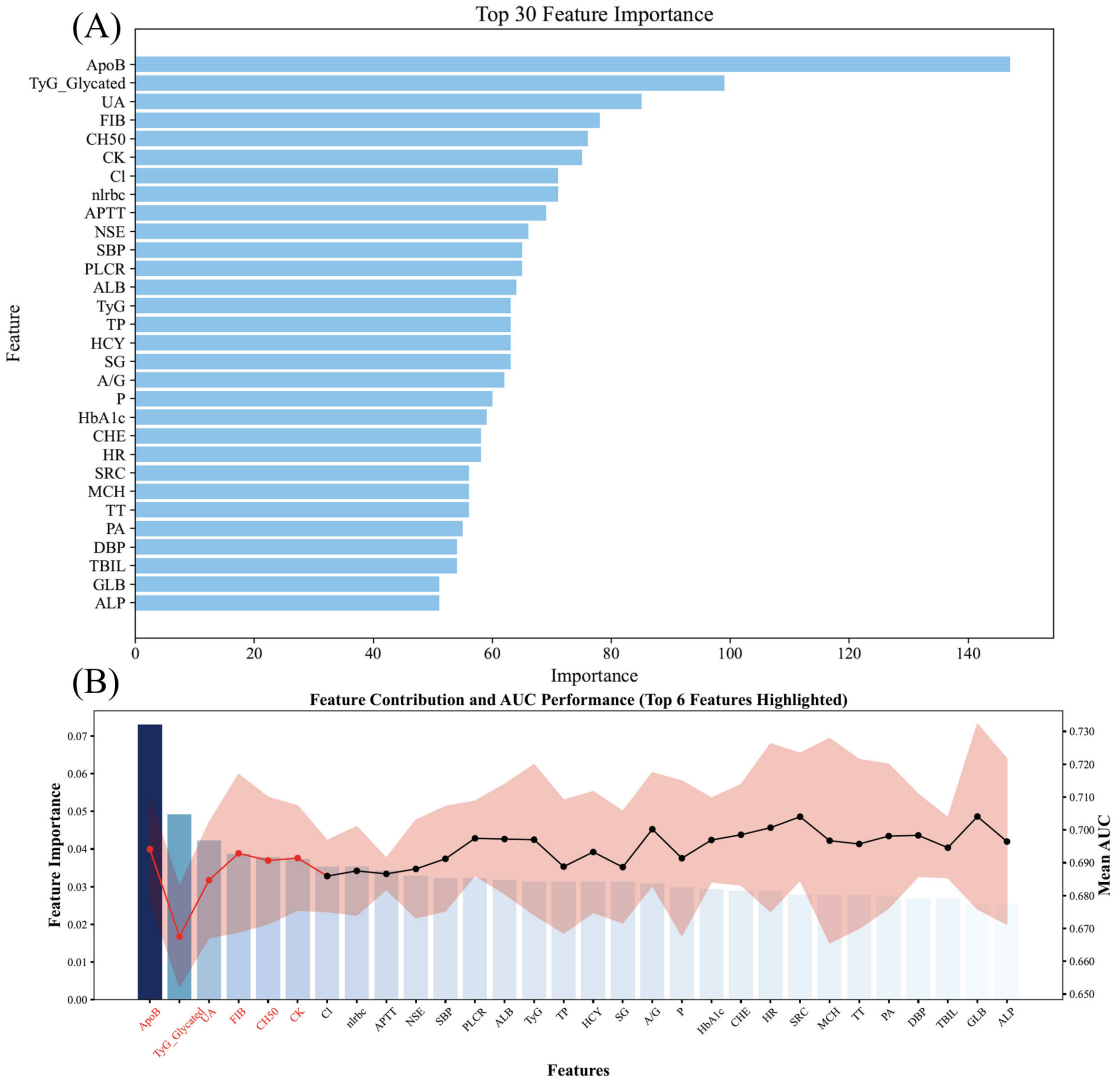
Variable selection via the LGBM model. **(A)** Variable importance ranking. **(B)** Contribution of variables to AUC evaluation via the LGBM classifier.

The dataset was randomly split into training (70%) and testing (30%) sets. Variable importance ranking by LGBM initially identified the top 30 predictors, with model performance plateauing after the top six (ApoB, gTyG, UA, FIB, CH50, CK), which were ultimately selected for model development (**Figure 3**).

**Figure 4:**
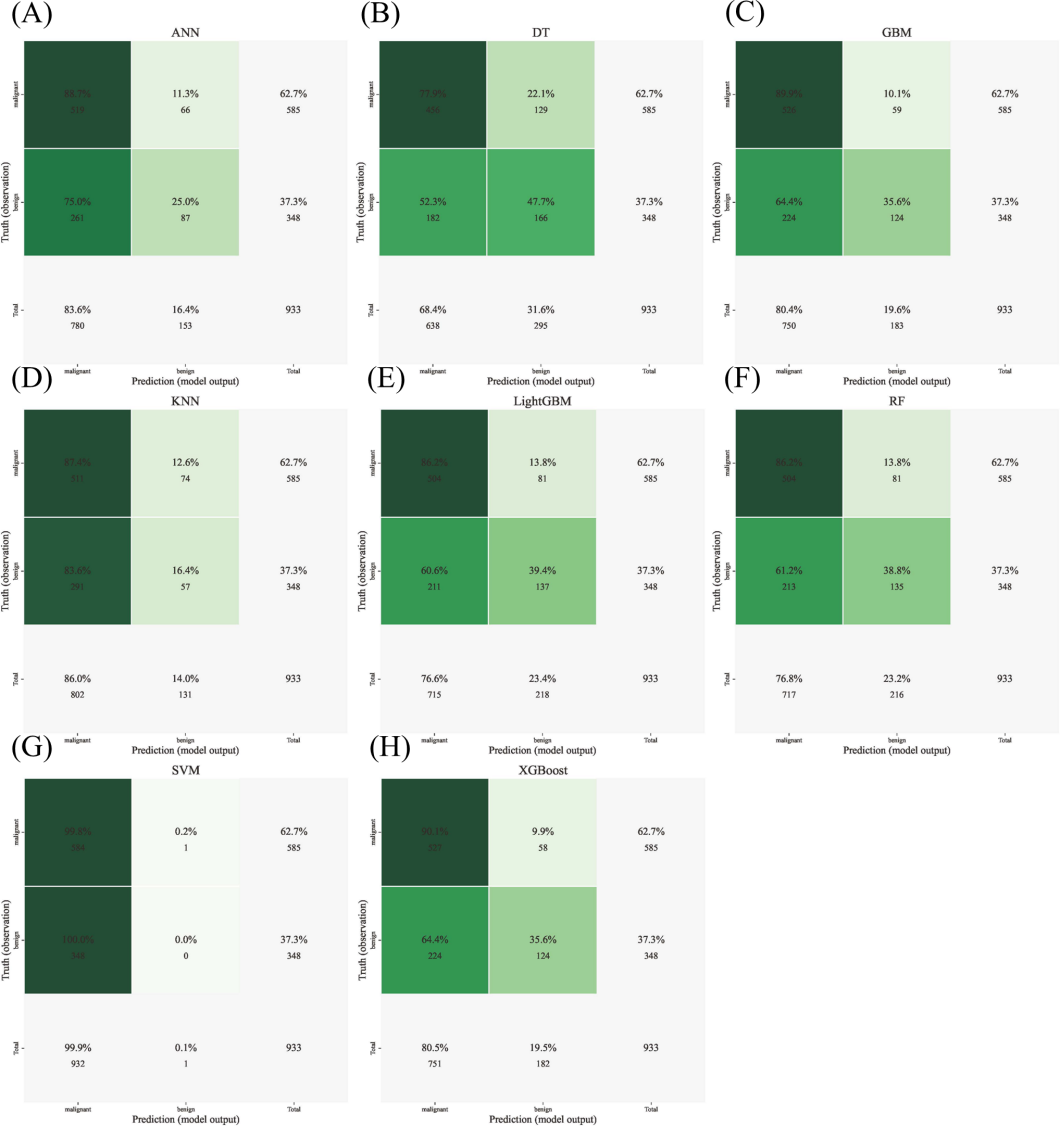
Confusion matrices of eight ML models. **(A)** Confusion matrix for the ANN. **(B)** Confusion matrix for DTs. **(C)** Confusion matrix for GBM. **(D)** Confusion matrix for KNN. **(E)** Confusion matrix for LGBM. **(F)** Confusion matrix for RF. **(G)** Confusion matrix for SVM. **(H)** Confusion matrix for XGBoost.

Eight algorithms were developed—ANN, DT, GBM, KNN, LGBM, RF, SVM, and XGB—with model-specific hyperparameters optimized via GridSearchCV and 10-fold cross-validation to ensure stability. Confusion matrices for each model are provided in **Figure 4** to visualize concordance between predicted and true labels.

**Figure 5:**
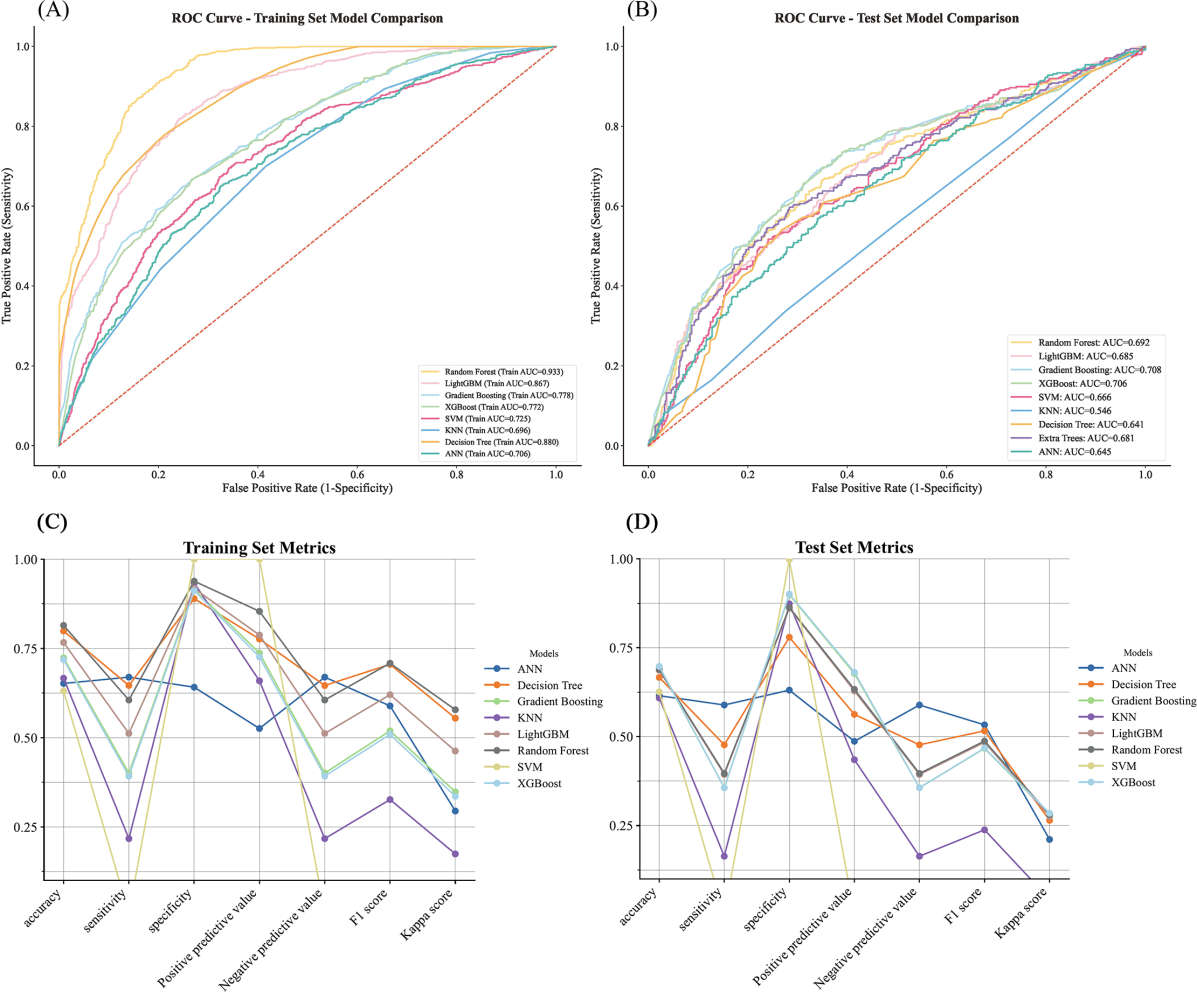
Comparison of eight ML models on training and test sets. **(A)** AUC values on the training set. **(B)** AUC values on the test set. **(C)** Line chart of evaluation metrics on the training set. **(D)** Line chart of evaluation metrics on the test set.

RF exhibited the best performance, with an AUC of 0.933 in the training set and high classification accuracy (0.814), sensitivity (0.939), specificity (0.939), PPV (0.854), and F1 score (0.709). LGBM, ANN, and XGB also performed well (AUC > 0.88), while DT and KNN showed lower predictive power. Full performance metrics are shown in **Figure 5** and **Supplementary Table 3-4**.

### 3.6 Model interpretation with SHAP

**Figure 6:**
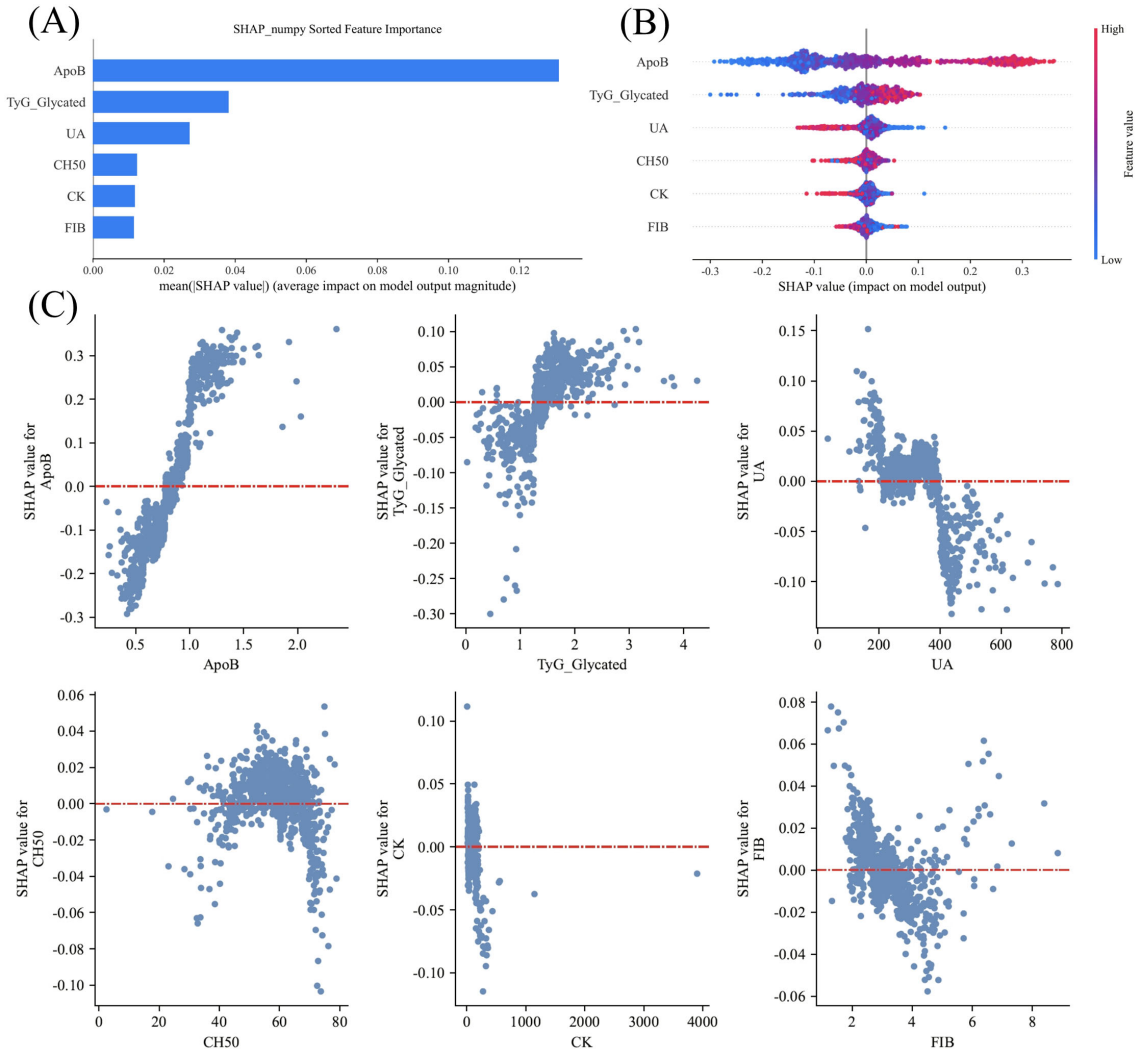
Global model interpretation via the SHAP method. **(A)** Bar chart of variable contributions. **(B)** Swarm plot. **(C)** Scatter plot. Each point represents a patient, with colors indicating feature values (red = high, blue = low). ApoB and gTyG contributed most strongly to prediction, indicating their pivotal roles in the model.

SHAP analysis revealed that ApoB and gTyG were the strongest contributors to model predictions (**Figure 6**). Higher levels of these biomarkers were associated with increased predicted risk of depression.

**Figure 6A** shows a bar chart of the SHAP values used to quantify and visualize the contribution of each variable to the model predictions. SHAP values represent the influence of each feature on the final prediction under different combinations of variables. Higher SHAP values indicate a greater impact of that variable on the model’s prediction. The contributions of each variable, ranked from largest to smallest, are as follows: ApoB, gTyG index, UA, CH50, CK, and FIB. The results show that ApoB is the core predictor for depression in CHF patients, while the gTyG index contributes significantly to the model’s predictive ability, ranking second. UA contributes third, whereas CH50, CK, and FIB have relatively smaller contributions to the model’s prediction ability.

**Figure 6B** displays a swarm plot, which shows the distribution of SHAP values for each feature. This visually represents how each feature influences the model’s prediction across different value ranges. SHAP values are color-coded (blue represents lower feature values, purple represents higher feature values) to reveal the relationship between feature values and prediction outcomes. Among all the features, ApoB has the greatest influence on SHAP, with higher values (shown in purple) corresponding to higher SHAP values, indicating a stronger impact of higher ApoB levels on the model’s prediction. The gTyG index is also an important feature, with a broad SHAP value distribution. Higher gTyG index values increase the model’s predicted output. Features such as UA, CH50, CK, and FIB contribute less to the model’s prediction. These results suggest that the model relies primarily on ApoB and gTyG index-related indicators, whereas factors such as UA, CH50, CK, and FIB have a lesser impact.

**Figure 6C** presents scatter plots of the SHAP values for each feature, revealing how each feature influences the model’s prediction output across different value ranges. As shown in the figure, increasing values of the ApoB and gTyG indices had a significant positive effect on the model’s risk prediction, indicating that higher values of these features are typically associated with a greater risk of CKD. In contrast, UA and CK were negatively correlated, whereas CH50 and FIB exhibited an approximately U-shaped relationship.

Representative individual waterfall plots show that the ApoB and gTyG indices are the primary factors influencing the occurrence of depression in CHF patients. As the levels of ApoB and the gTyG index increase, the risk of depression also increases (**Supplementary Figure 2**).

### 3.7 Web-based prediction tool

A web application integrating the RF model and SHAP interpretation was developed to provide individualized risk prediction (http://dpm.segsage.cn/. Accessed 31 December 2025). Users can input ApoB, gTyG, and clinical parameters to obtain risk estimates with explanatory features. The interface is shown in **Supplementary Figure 3**.

## 4 Discussion

In this study, we developed and evaluated several ML models to predict depression risk in patients with CHF using key biomarkers such as ApoB, the gTyG index, and other clinical features. Our results revealed that higher levels of the ApoB and gTyG indices were significantly associated with an increased risk of depression, with odds ratios for ApoB Q2, Q3, and Q4 being 1.53, 3.00, and 7.90 times higher than those of the Q1 group, respectively (P < 0.001). The RF model achieved the best performance, with an AUC of 0.933 in the training set. SHAP analysis revealed that the ApoB and gTyG indices were the most important predictors, with higher levels correlating with a greater risk of depression. These findings emphasize the utility of ML in predicting depression risk in CHF patients and suggest that the ApoB and gTyG indices could serve as important biomarkers for early detection and intervention.

Mounting evidence suggests that depression is correlated with altered lipid metabolism, increased inflammation, disrupted neuro-immuno-endocrine balance and oxidative stress (29, 30). Studies have reported consistently increased levels of inflammatory markers, such as elevated IL-6, CRP, IL-1, and TNF-α, in depressed patients (31, 32). Oxidative stress, as evidenced by increased malondialdehyde and 8-hydroxy-2′-deoxyguanosine levels, together with reduced antioxidant levels, is also related to depression (29, 31). A pooled analysis of 230 metabolic markers in patients with depression revealed an atherogenic lipid profile in depression, marked by increased very low‐density lipoprotein, triglycerides, and ApoB alongside decreased high‐density lipoprotein (33). Reviews and primary studies further revealed that major depression, including glucocorticoid resistance, elevated cortisol, and shifts in the tryptophan-kynurenine pathway that may promote neurodegenerative processes, is associated with neuroimmunoendocrine imbalances (34).

In the context of CHF, a growing body of evidence suggests that alterations in lipid metabolism are linked to disease prognosis and multiple complications (35). Each atherogenic particle carries one ApoB molecule; thus, ApoB is a direct measure of the total number of these particles in circulation (36, 37). It has been recognized in multiple guidelines as a superior marker for residual cardiovascular risk, especially in patients with metabolic syndrome and diabetes (38).

In addition to its cardiovascular implications, recent studies have revealed associations between ApoB and psychiatric conditions, particularly depression. Bot et al. conducted a large-scale metabolomic analysis and reported that higher levels of ApoB were significantly associated with an increased risk of depression (33). Another study by Hui et al. reported that elevated ApoB levels in patients with major depressive disorder were linked to cognitive deficits, suggesting that metabolic pathways shared between depression and dyslipidemia (39). However, studies specifically exploring the relationship between ApoB and CHF remain limited.

ApoB levels are related to depression in various ways. In adult samples in general, studies have reported that higher ApoB is linked to greater depressive severity (33, 40). One study reported that depression predicts an increase in ApoB, and another reported that increased ApoB increases the odds of depression. In a sample of Han Chinese depressed patients, higher ApoB correlated with poorer delayed memory. In contrast, a study of middle-aged Finnish men revealed no significant difference in ApoB levels between depressed and nondepressed groups, whereas among elderly individuals with mild cognitive impairment, lower ApoB levels are associated with depressive symptoms (41). Mechanistically, ApoB-containing atherogenic lipoproteins may promote endothelial dysfunction and microvascular injury, amplify systemic inflammation and lipid peroxidation, and perturb monoaminergic signaling and neuroplasticity—pathways long implicated in depression. Our model outputs are concordant with these mechanisms, suggesting that in CHF patients, ApoB may function as a metabolic-vascular signal of depressive vulnerability (39, 40).

Recent studies have explored the association between the TyG index and depression, as well as its relationship with CHF. A substantial cross-sectional study established that elevated TyG index indices were demonstrably associated with heightened depressive symptoms in a sample of U.S. adults (42). A meta-analysis of six cross-sectional studies revealed that a high TyG index was associated with a higher incidence of depression in adults (43). Moreover, the TyG index was associated with an elevated risk of CHF in a large cohort study, with a J-shaped dose–response relationship being observed (44). These findings suggest that the TyG index may serve as a useful tool for identifying individuals at risk for both depression and CHF.

The TyG index is a pragmatic surrogate of insulin resistance with good agreement with the hyperinsulinemic-euglycemic clamp (45) and shows positive correlations with HbA1c (46). Fasting glucose fluctuates with stress and medications, whereas HbA1c reflects chronic glycemic exposure and is more stable and comparable across settings (47). We therefore combined TG with HbA1c to construct the gTyG index, aiming to capture the chronic glyco-lipid/IR burden more robustly than TG or glucose alone. The results revealed that the gTyG index outperformed other single metabolic surrogates, indicating that an IR-inflammation-neuroendocrine pathway is involved in depression in CHF patients.

Recently, ML techniques have demonstrated improved predictive accuracy in various cardiovascular applications, including CHF phenotyping and outcome prediction. However, only a few studies have applied ML methods to psychiatric risk modeling in cardiovascular diseases. Nowakowska et al. examined four ML methods and reported that ML models, particularly the RF model, showed a moderate ability to predict depression risk in patients undergoing coronary artery bypass graft surgery via biomarker data, especially soluble receptor for advanced glycation end products (sRAGE) (48).

The model under discussion facilitates the screening and stratified management of CHF patients and can be used as an interpretable, individualized decision-making tool in clinical practice, demonstrating both usability and scalability. In the context of CHF follow-up, the integration of simple thresholds for the ApoB and gTyG indices can facilitate the identification of individuals at high risk for depression at an early stage. This, in turn, can initiate standardized depression assessment and intervention pathways, which may enhance CHF patients’ adherence to treatment, reduce the risk of readmission, and mitigate mortality risk (5). SHAP-based individual explanations facilitate risk communication and alignment with lipid-lowering and insulin-sensitizing strategies when appropriate. ApoB, TG, HbA1c, and other indicators are widely available test indicators that can be detected in the majority of primary care hospitals. The gTyG index is a readily calculable metric that can be incorporated into electronic medical records for automated calculation and is conducive to expeditious deployment.

The marginal gains of the other inflammatory, oxidative stress and conventional biochemical indicators included in our cohort were limited. They failed to deliver significant improvements in multimodel comparisons and validation. Given the heterogeneity of CHF patients and their multiple comorbidities, the effects of the above indicators may be diluted by factors such as medication, infection, and acute decompensation, resulting in a poor correlation.

Beyond metabolic and inflammatory pathways, structural thoracic characteristics and associated cardiac variations may also contribute to psychological vulnerability in CHF. Systematic reviews report that thoracic skeletal abnormalities, such as reduced antero–posterior chest diameter, pectus excavatum, pectus carinatum, scoliosis, and straight-back morphology, can alter cardiopulmonary mechanics and influence cardiac positioning (49). These anatomical variations may promote mechanical distortion of the mitral annulus and subvalvular structures, increasing susceptibility to mitral valve prolapse. Meta-analytic evidence shows that mitral valve prolapse is more common among individuals with anxiety-related disorders than in the general population, supporting links between valvular biomechanics, autonomic regulation, and affective symptoms (50). Clinical manifestations typically associated with mitral valve prolapse, including palpitations, atypical chest discomfort, and dyspnea, may further reinforce anxiety or depressive tendencies through both physiological responses and heightened symptom perception. Although our study did not evaluate thoracic morphology or valvular structure, their potential coexistence with metabolic biomarkers cannot be excluded.

### 4.1 Limitations

The present study adopted a single-center, retrospective research design, modeling baseline features only, without longitudinal dynamics. The external generalizability of this study’s findings is limited insofar as causal inference may be subject to selection and information bias.

The cutoff thresholds used to classify high and low risk patients were derived separately in the training and validation cohorts, which may reduce interpretability and limit the stability of risk stratification across different populations. The absence of a unified threshold also constrains clinical applicability, as consistent cutoff values are essential for reliable decision-making. Methodological guidance from previous work further underscores the importance of evaluating threshold stability in external cohorts to enhance reproducibility and clinical utility (51). Future studies with larger, independent cohorts are therefore needed to validate consistent cutoff strategies and improve generalizability.

Although ApoB and the gTyG index were identified as key predictors of depression risk in CHF patients, the biological or pathway-level relationships between these biomarkers remain unclear. How metabolic, inflammatory, and insulin-resistance–related processes jointly contribute to depressive vulnerability cannot be determined from the routinely collected clinical indicators used in this study, which lack molecular or multi-omic information needed to reconstruct mechanistic pathways or identify intermediate mediators. Future studies may consider pathway-oriented analytic frameworks that characterize structured relationships among biological variables and allow investigation of potential direct and indirect effects. Prior research has applied conceptually similar approaches to delineate structured associations among multiple predictors, providing a methodological reference for future mechanistic exploration (52).

Furthermore, the present study was subject to uncontrolled confounders, including the bidirectional effects of antidepressants, statins, SGLT2i/GLP-1RA, and other drugs on metabolism and mood. Multicenter external validation, prospective time-updated modeling, and interventional studies are warranted to test whether an ApoB plus TyG-glycated–guided strategy improves depressive and CHF outcomes.

## 5 Conclusion

This study aimed to develop and validate ML models for predicting depression in patients with CHF. Our results demonstrate that biomarkers such as the ApoB and gTyG indices play critical roles in predicting depression risk in CHF patients. Among the various ML models evaluated, the RF model has emerged as the most effective tool for identifying patients at high risk for depression.

Despite including a range of inflammatory, oxidative stress, and conventional biochemical markers, the ApoB and gTyG indices showed significant predictive power. Other markers, including UA, FIB and CK, failed to substantially improve model performance. These findings suggest that factors such as medication, infection, and acute decompensation may dilute the impact of additional biomarkers, reducing their relevance in depression prediction.

By integrating biological, psychological, and computational domains, our study addresses a critical gap in CHF management. These findings offer a potential approach to risk stratification and early mental health intervention, ultimately enhancing the ability to identify at-risk patients and improve their clinical outcomes.

## Data Availability

The original contributions presented in the study are included in the article/**Supplementary Material**. Further inquiries can be directed to the corresponding author.
