# Supplementary material for: Biomarker-based depression risk prediction in chronic heart failure patients: an interpretable machine learning approach
Source: Front Endocrinol (Lausanne). 2025 Dec 11;16:1737713. doi: 10.3389/fendo.2025.1737713 (PMC12738324; doi:10.3389/fendo.2025.1737713)
Supplement: Supplementary file 1 [file DataSheet1.docx]

Supplementary Material

# Supplementary Data

None.

# Supplementary Figures and Tables

## Supplementary Figures





**Supplementary Figure 1 Flowchart of patient selection.** Of 8,645 initially screened patients with CHF, 3,110 were included after excluding those with missing data. Final cohort: 1,159 with depression and 1,951 controls.


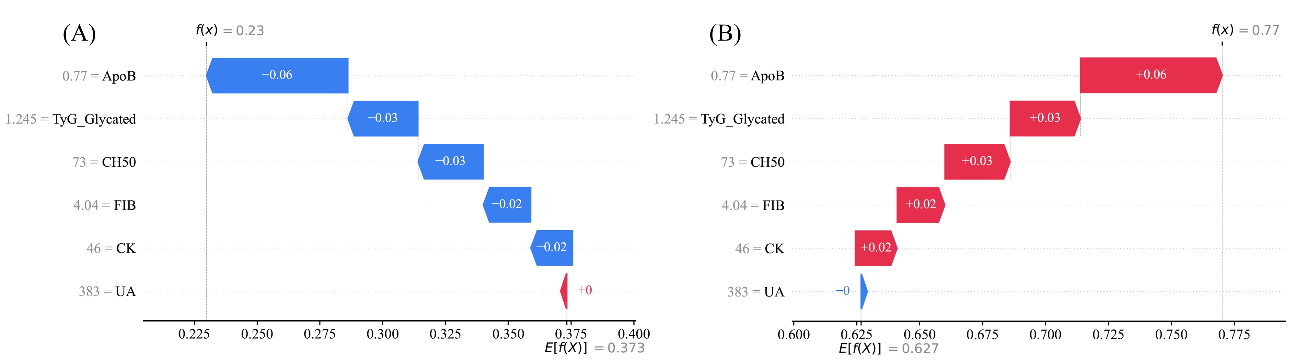


**Supplementary Figure 2. Representative SHAP waterfall plots for individual cases.** Waterfall plots showing how ApoB, gTyG, and other top predictors contribute to the predicted risk for two representative patients (depressed vs. nondepressed). Positive SHAP values indicate risk-enhancing effects, and negative values indicate risk-reducing effects.


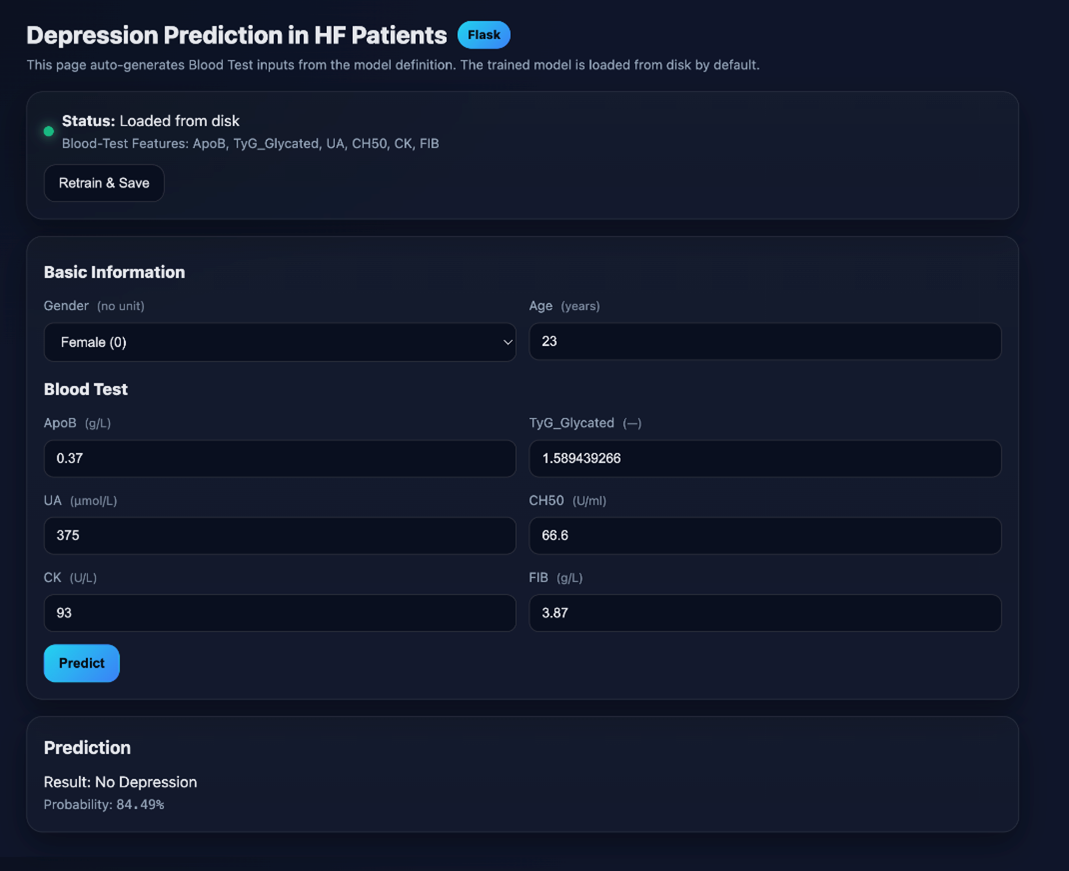


**Supplementary Figure 3 Web-based risk prediction tool interface.** Screenshot of the interactive web application for individualized depression risk prediction. Users can input ApoB, gTyG, and clinical parameters to obtain real-time risk estimates with SHAP-based explanations.

## Supplementary Tables

**SupplementaryTable 1** Risk Ratios of Depression across gTyG Index Levels in Different Models

| Variables | Model1 | |  | Model2 | |  | Model3 | |
| --- | --- | --- | --- | --- | --- | --- | --- | --- |
|  | OR (95%CI) | *P* |  | OR (95%CI) | *P* |  | OR (95%CI) | *P* |
| gTyG group |  |  |  |  |  |  |  |  |
| 1 | 1.00 (Reference) |  |  | 1.00 (Reference) |  |  | 1.00 (Reference) |  |
| 2 | 1.65 (1.33 ~ 2.05) | **<.001** |  | 1.50 (1.14 ~ 1.97) | **0.004** |  | 1.48 (1.11 ~ 1.97) | **0.008** |
| 3 | 1.99 (1.60 ~ 2.47) | **<.001** |  | 1.94 (1.42 ~ 2.63) | **<.001** |  | 1.93 (1.38 ~ 2.69) | **<.001** |
| 4 | 2.81 (2.26 ~ 3.48) | **<.001** |  | 2.83 (1.89 ~ 4.22) | **<.001** |  | 2.88 (1.88 ~ 4.41) | **<.001** |
| OR: Odds Ratio, CI: Confidence Interval  Model1: Crude  Model2: Adjust: Hypertension, CHD, Stroke, ApoB, TyG index, Age, HR, DBP, UA, CHO, TP, GLB, TG, CHE  Model3: Adjust: Hypertension, CHD, Diabetes, Stroke, OP, Anemia, ApoB, TyG index, Age, HR, DBP, UA, CHO, TP, CH50, SG, Cl, LYMPH, GLB, TG, ALB, P, HbA1c, RBC, HCT, FIB, CHE, PLT, PCT, HCY, HGB | | | | | | | | |

**Supplementary Table 2** Risk Ratios of Depression across TyG Index Levels in Different Models

| Variables | Model1 | |  | Model2 | |  | Model3 | |
| --- | --- | --- | --- | --- | --- | --- | --- | --- |
|  | OR (95%CI) | *P* |  | OR (95%CI) | *P* |  | OR (95%CI) | *P* |
| TyG index group |  |  |  |  |  |  |  |  |
| 1 | 1.00 (Reference) |  |  | 1.00 (Reference) |  |  | 1.00 (Reference) |  |
| 2 | 1.55 (1.25 ~ 1.92) | **<.001** |  | 1.16 (0.90 ~ 1.50) | 0.247 |  | 1.19 (0.92 ~ 1.55) | 0.191 |
| 3 | 1.91 (1.55 ~ 2.37) | **<.001** |  | 1.30 (0.99 ~ 1.72) | 0.063 |  | 1.37 (1.02 ~ 1.84) | **0.037** |
| 4 | 2.14 (1.73 ~ 2.65) | **<.001** |  | 1.02 (0.70 ~ 1.48) | 0.935 |  | 1.09 (0.74 ~ 1.61) | 0.658 |
| OR: Odds Ratio, CI: Confidence Interval | | | | | | | | |
| Model1: Crude | | | | | | | | |
| Model2: Adjust: Hypertension, CHD, Stroke, ApoB, gTyG, Age, HR, DBP, UA, CHO, TP, GLB, TG, CHE | | | | | | | | |
| Model3: Adjust: Hypertension, CHD, Diabetes, Stroke, OP, Anemia, ApoB, gTyG, Age, HR, DBP, UA, CHO, TP, CH50, SG, Cl, LYMPH, GLB, TG, ALB, P, HbA1c, RBC, HCT, FIB, CHE, PLT, PCT, HCY, HGB | | | | | | | | |

**Supplementary Table 3** Model evaluation metrics for the training set

| **Metrics** | **ANN** | **Decision Tree** | **Gradient Boosting** | **KNN** | **LightGBM** | **Random Forest** | **SVM** | **XGBoost** |
| --- | --- | --- | --- | --- | --- | --- | --- | --- |
| accuracy | 0.652 | 0.799 | 0.723 | 0.667 | 0.767 | 0.814 | 0.631 | 0.718 |
| sensitivity | 0.67 | 0.646 | 0.401 | 0.217 | 0.512 | 0.605 | 0.009 | 0.392 |
| specificity | 0.641 | 0.889 | 0.915 | 0.933 | 0.918 | 0.939 | 1 | 0.912 |
| PPV | 0.526 | 0.776 | 0.737 | 0.659 | 0.787 | 0.854 | 1 | 0.726 |
| NPV | 0.67 | 0.646 | 0.401 | 0.217 | 0.512 | 0.605 | 0.009 | 0.392 |
| F1 score | 0.589 | 0.705 | 0.519 | 0.327 | 0.62 | 0.709 | 0.017 | 0.509 |
| Kappa score | 0.294 | 0.554 | 0.348 | 0.174 | 0.463 | 0.578 | 0.011 | 0.336 |

**Supplementary Table 4** Model evaluation metrics for the testing set

| **Metrics** | **ANN** | **Decision Tree** | **Gradient Boosting** | **KNN** | **LightGBM** | **Random Forest** | **SVM** | **XGBoost** |
| --- | --- | --- | --- | --- | --- | --- | --- | --- |
| accuracy | 0.615 | 0.667 | 0.697 | 0.609 | 0.687 | 0.689 | 0.626 | 0.698 |
| sensitivity | 0.589 | 0.477 | 0.356 | 0.164 | 0.394 | 0.397 | 0 | 0.356 |
| specificity | 0.631 | 0.779 | 0.899 | 0.874 | 0.862 | 0.863 | 0.998 | 0.901 |
| PPV | 0.487 | 0.563 | 0.678 | 0.435 | 0.628 | 0.633 | 0 | 0.681 |
| NPV | 0.589 | 0.477 | 0.356 | 0.164 | 0.394 | 0.397 | 0 | 0.356 |
| F1 score | 0.533 | 0.516 | 0.467 | 0.238 | 0.484 | 0.488 | 0 | 0.468 |
| Kappa score | 0.211 | 0.265 | 0.283 | 0.043 | 0.276 | 0.281 | -0.002 | 0.285 |
